# The relationship between inclusive leadership in sustainable education and teachers' subjective wellbeing in Türkiye: the mediating role of affective commitment

**DOI:** 10.3389/fpsyg.2025.1689020

**Published:** 2025-12-12

**Authors:** Mehmet Nezir Çevik

**Affiliations:** Department of Religious Education, Faculty of Theology, Siirt University, Siirt, Türkiye

**Keywords:** inclusive leadership, subjective wellbeing, affective commitment, mediating role, sustainable education

## Abstract

**Introduction:**

Supporting teachers' subjective wellbeing in schools is a critical factor that directly is associated with both teacher productivity and student achievement. However, the role of inclusive leadership and possible mediating mechanisms in this context has not been sufficiently investigated. The present study examines the association between school administrators' inclusive leadership behaviors and teachers' subjective wellbeing and the mediating role of affective commitment in this relationship.

**Methods:**

Data was collected online from 585 teachers working at different levels in four regions of Türkiye. Measurements were taken using the Inclusive Leadership Scale, Affective Commitment Subscale, and Subjective Wellbeing Scale. The Structural Equation Model (SEM) was used for data analysis in the study, and the significance of direct and indirect associations between variables was evaluated using the bootstrap method. The measurement and structural models showed acceptable fit (χ^2^/df = 3.46; CFI = 0.92; TLI = 0.91; RMSEA = 0.065).

**Results:**

According to the results, inclusive leadership was positively associated with teachers' subjective wellbeing both directly and indirectly through affective commitment. Inclusive leadership had a moderate direct effect on subjective wellbeing (β = 0.51), while affective commitment had a weaker but significant effect (β = 0.17). Inclusive leadership strongly predicted affective commitment (β = 0.85). The indirect effect was significant (β = 0.14), with a VAF of approximately 22%, indicating partial mediation. Overall, the model explained 72% of the variance in affective commitment and 32% of the variance in subjective wellbeing.

**Discussion:**

The findings of the study reveal that inclusive leadership is significantly associated with teachers‘ subjective wellbeing. Practically, these results highlight the importance of leadership practices that foster transparency, accessibility, participation, and valuing teacher input. Integrating inclusive leadership principles into leadership training programmes and educational policy frameworks may strengthen teachers' motivation, organizational commitment, and wellbeing. Such practices may also reduce stress and burnout among teachers, contributing to more sustainable and supportive school environments. Future research may further examine these associations across different school types and cultural contexts.

## 1 Introduction

The question of how teachers working in schools, which are intellectually, emotionally and physically challenging environments, can maintain their mental, emotional and professional integrity has been debated in many countries for many years (Goodlad, 2004). These discussions have drawn researchers' attention to the concept of subjective wellbeing, revealing that it plays a decisive role in making teachers' professional lives sustainable (Song et al., 2020). The interest in teachers' subjective wellbeing stems not only from the intense workload inherent in the profession but also from research showing that society has high expectations of teachers (Day, 2017; Huberman, 1993). On the other hand, teachers' subjective wellbeing is also of great importance for individual development and organizational success (Cao et al., 2022). Indeed, previous studies (e.g., Rogozińska-Pawełczyk, 2023; Song et al., 2020; Wang et al., 2022) further support this view by showing that factors such as psychological wellbeing, job satisfaction, affective commitment, inclusiveness, and leadership are associated with teachers' subjective wellbeing. Among the factors shaping subjective wellbeing in the teaching profession, the literature often emphasizes the decisive role of affective commitment and leadership variables (Xu and Pang, 2024). Recent empirical studies indicate that subjective wellbeing is shaped not merely by broad psychological tendencies, but by specific organizational and relational dynamics within schools (Cao et al., 2022; Xu and Pang, 2024). For example, (Rogozińska-Pawełczyk 2023) identified positive associations between inclusive leadership, psychological contract fulfillment and employees' workplace and life-related wellbeing, underscoring the role of leadership in shaping day-to-day experiences at work, while Wang et al. (2022) highlighted that teachers' subjective wellbeing is influenced by both personal resources such as self-efficacy and resilience and the broader environmental conditions of their work context. Similarly, recent research in the Turkish context has emphasized that leadership behaviors significantly influence teachers' psychological states, yet the underlying mechanisms remain insufficiently explained (Çevik and Dogan, 2025). In addition, prior work has shown that leaders' emotional feedback patterns shape the quality of leader-member exchange in schools, demonstrating the emotional foundations of leadership processes (Berkovich and Eyal, 2021). Despite these findings, empirical research examining how emotional factors such as affective commitment operate together with leadership practices within a single integrated model remains limited (Çelik et al., 2024; Liu et al., 2024). This gap highlights the need for studies that investigate how organizational conditions and emotional mechanisms jointly influence teachers' subjective wellbeing.

Teachers‘ affective commitment to their profession, beyond an individual emotional state, emerges as a fundamental element that strengthens relationships in the school environment and supports teachers' subjective wellbeing (Shu, 2022). Therefore, increasing teachers‘ affective commitment has become a central topic in education research worldwide (Amoah et al., 2021). In other words, emotions play a decisive role in both the teaching process and teachers' professional lives, making teaching a profession that involves intense emotional interactions (Hargreaves, 1998). In particular, the presence of a supportive leadership approach strengthens teachers‘ affective commitment to the school, increasing their subjective wellbeing and contributing to the creation of a positive school climate (Wang et al., 2021). In this context, contextual factors, together with affective commitment, are associated with teachers' subjective wellbeing by shaping their attitudes and behavioral outcomes (Li et al., 2024). Given these emotional dynamics, it is important to consider how affective commitment is connected with broader organizational conditions such as leadership behaviors.

Unlike affective commitment, various leadership practices are meaningfully associated with teachers' subjective wellbeing (Choi et al., 2017). For example, Arnold et al. (2007) argued that transformational leadership increases employee wellbeing by revealing the meaning of work. Braun and Peus (2018) found a positive relationship between authentic leadership and employee job satisfaction. At the same time, different leadership styles play a key role in their environment (Randel et al., 2016) by creating an environment that values diversity within the organization and contributes to the formation of an inclusive atmosphere (Ashikali et al., 2021). In fact, inclusive leadership is a leadership approach that supports subjective wellbeing by providing a high level of affective commitment and satisfaction in the work environment (Brimhall and Mor Barak, 2018). In other words, when employees perceive leadership in an environment where differences are valued, their affective commitment to the organization increases and their subjective wellbeing levels rise (Çelik et al., 2024). Therefore, school principals who demonstrate inclusive leadership can contribute to the development of a sustainable educational culture by supporting diversity and participation (Ye et al., 2019).

Given the conceptual relevance of sustainable education to leadership practices, it is important to clarify how sustainability is approached in the present study. In the context of this study, sustainable education is discussed as a guiding framework rather than an empirically tested variable. Sustainable education requires a holistic approach that goes beyond environmental management and economic feasibility to include the promotion of social justice (Barth et al., 2015). This is because supporting teachers' affective commitment and subjective wellbeing is conceptually aligned with the creation of a sustainable education culture (Rogozińska-Pawełczyk, 2023). In this respect, inclusive leadership in educational organizations is conceptually linked to affective commitment and subjective wellbeing by creating an environment that values differences and makes employees feel safe and accepted (Brimhall and Mor Barak, 2018; Wang et al., 2021). The goal of sustainable education to reduce social inequalities aligns strongly with the inclusive leadership approach, ensuring the effective management of differences and contributing to a broader understanding of sustainability within educational contexts (Artamonova et al., 2019; Ye et al., 2019). Although various leadership approaches have been found to be positively related to subjective wellbeing in the literature (Bogler and Berkovich, 2022; Ortiz-Gómez et al., 2022; Zhang et al., 2023), the effect of inclusive leadership in this context has been relatively limited (Xu and Pang, 2024). Similarly, the effect of inclusive leadership on teachers' subjective wellbeing in the context of sustainable education remains unclear. Although the direct associations between leadership, affective commitment and subjective wellbeing have been widely acknowledged in the existing literature (e.g., Choi et al., 2017; Brimhall and Mor Barak, 2018; Wang et al., 2021), relatively fewer studies have investigated how these variables operate together within a single mediation framework. For instance, (Brimhall and Mor Barak 2018) demonstrated that inclusive leadership enhances employees' sense of belonging and affective commitment, which in turn contributes to higher levels of wellbeing—suggesting a potential mediating process. Similarly, Liu et al. (2024) showed that inclusive leadership predicts wellbeing through psychological need satisfaction, providing further evidence that emotional mechanisms may serve as indirect pathways linking leadership to positive employee outcomes. However, studies explicitly examining affective commitment as the mediating mechanism connecting inclusive leadership to teachers' subjective wellbeing, particularly within school contexts, remain scarce (Çelik et al., 2024). In response to this gap, the present study places the mediation mechanism at the center of its theoretical contribution. By testing a model in which inclusive leadership predicts teachers' subjective wellbeing both directly and indirectly through affective commitment, the study extends previous work by empirically evaluating a theoretically implied but under examined pathway. Furthermore, conducting this investigation in the Turkish education system, which is marked by strong centralization, heavy administrative expectations and increasing diversity, provides additional contextual value, as most mediation-based leadership wellbeing studies have been carried out in less centralized or more stable settings.

Türkiye's education system is highly centralized, with key decisions regarding curriculum, assessment and personnel policies largely determined at the national level. This centralized and bureaucratic structure, shaped by long-standing administrative traditions and societal norms, has been extensively discussed in the Turkish educational leadership literature (Bellibaş and Kilinç, 2023; Kilinç et al., 2021). When combined with large class sizes and high-stakes examinations, this structure places considerable pressure on teachers and school leaders and restricts their autonomy in school-level decision-making (Toprak et al., 2022). In addition to these structural pressures, Türkiye has experienced a substantial increase in student diversity due to regional conflicts, particularly the Syrian civil war, which has led millions of displaced individuals to seek refuge in the country. As a result, public schools now serve large numbers of refugee and immigrant students, creating classrooms characterized by significant cultural, linguistic, and socio-economic diversity. Studies show that school leaders frequently encounter challenges such as language barriers, low academic achievement, absenteeism and adaptation difficulties among refugee students (Bruckauf, 2016; Edmonds and Flahault, 2021). To address these challenges and ensure equitable participation for all students, school principals engage in guidance and collaboration activities, organize cultural and sports events, develop adaptation and support programs, and provide interpreter assistance when needed, thereby demonstrating inclusive leadership practices (Culha and Nazli, 2023). These findings indicate that increasing diversity, influenced by regional humanitarian conditions, has significantly reinforced the importance of inclusive, adaptive, and culturally responsive leadership practices in Turkish schools.

Inclusive leadership not only strengthens team members‘ sense of belonging but also encourages them to behaviorally express their unique strengths (Randel et al., 2018). Being accepted by the group and being able to effectively utilize individual competencies are among the fundamental prerequisites for employees' subjective wellbeing (Diener et al., 2018). Sustainable education practices aimed at reducing social inequalities require leadership approaches that support diversity and participation to be effectively integrated into educational environments (Artamonova et al., 2019). Therefore, it is stated that inclusive leaders who can effectively manage differences play a decisive role in the formation of a sustainable education culture (Ye et al., 2019). Some researchers (e.g., Liu et al., 2024), while acknowledging that inclusive leadership supports subjective wellbeing, argue that this relationship is based on the fulfillment of intrinsic needs, which has a more lasting effect than extrinsic motivations and strengthens affective commitment. Despite these conceptual insights, there remains a need to empirically test how these mechanisms operate in specific educational contexts.

Identifying factors that strengthen emotional attachment in schools and implementing effective educational interventions in this regard is critical for improving teachers' subjective wellbeing (Heidari et al., 2022). Therefore, we hypothesized that emotional attachment could be an important mediating variable linking inclusive leadership and teachers' subjective wellbeing. Furthermore, the Turkish context provides an important research ground for understanding how inclusive leadership and teachers' subjective wellbeing affect affective commitment. This is because approximately 68% of teachers in Türkiye say they are considering leaving the teaching profession (Türk Eğitim-Sen, 2024). To solve this problem, policymakers aim to increase teachers' subjective wellbeing by strengthening their affective commitment to their institutions (Çetin, 2019). However, a review of the literature reveals that issues such as low job satisfaction, emotional exhaustion, and burnout have become more pronounced during the rapid education reform process (Toprakci and Yilmaz Çildir, 2025). This situation highlights the need to investigate leadership approaches that can increase the emotional commitment of teachers working in developing countries such as Türkiye and support their subjective wellbeing. Therefore, this study aims to make a meaningful contribution to the literature by examining the mediating role of affective commitment in the association between inclusive leadership and teachers' subjective wellbeing, within the contextual framework of sustainable education in Türkiye—a system characterized by centralized and bureaucratic structures. The study also seeks to address the following research questions.

1. Is inclusive leadership positively associated with teachers' subjective wellbeing and affective commitment levels?
2. Is affective commitment positively associated with teachers' subjective wellbeing?
3. Does affective commitment mediate the association between inclusive leadership and teachers' subjective wellbeing?

### 1.1 Research context and conceptual model

School policies in Türkiye are shaped by centralized structures, cultural values, and social change (Bellibaş and Kilinç, 2023). Within this structure, school administrators strive to create a school climate that is sensitive to cultural diversity, liberal, and egalitarian through inclusive leadership approaches, despite their limited authority (Culha and Nazli, 2023). This approach is particularly important in schools working with migrants and refugees (Gümüş and Buyukgoze, 2023). In Türkiye, teachers face multidimensional difficulties due to socio-cultural factors such as economic hardship, regional living conditions, working with migrant and refugee students, and extraordinary circumstances such as disasters (Çevik and Dogan, 2025; Selçuk and Güzel, 2025). Such challenges can negatively affect both teachers' professional performance and their subjective wellbeing levels (Kuru, 2023). In this context, it can be said that inclusive leadership plays an important role in protecting and strengthening teachers' subjective wellbeing by increasing affective commitment in the school environment. The current research can provide important insights for improving the education systems of developing countries such as Türkiye, which have centralized structures, cultural values, and social changes. In particular, the study discusses sustainability as a contextual consideration that helps explain the importance of inclusive leadership in promoting teachers' wellbeing, rather than as a directly measured construct.

**Figure 1:**
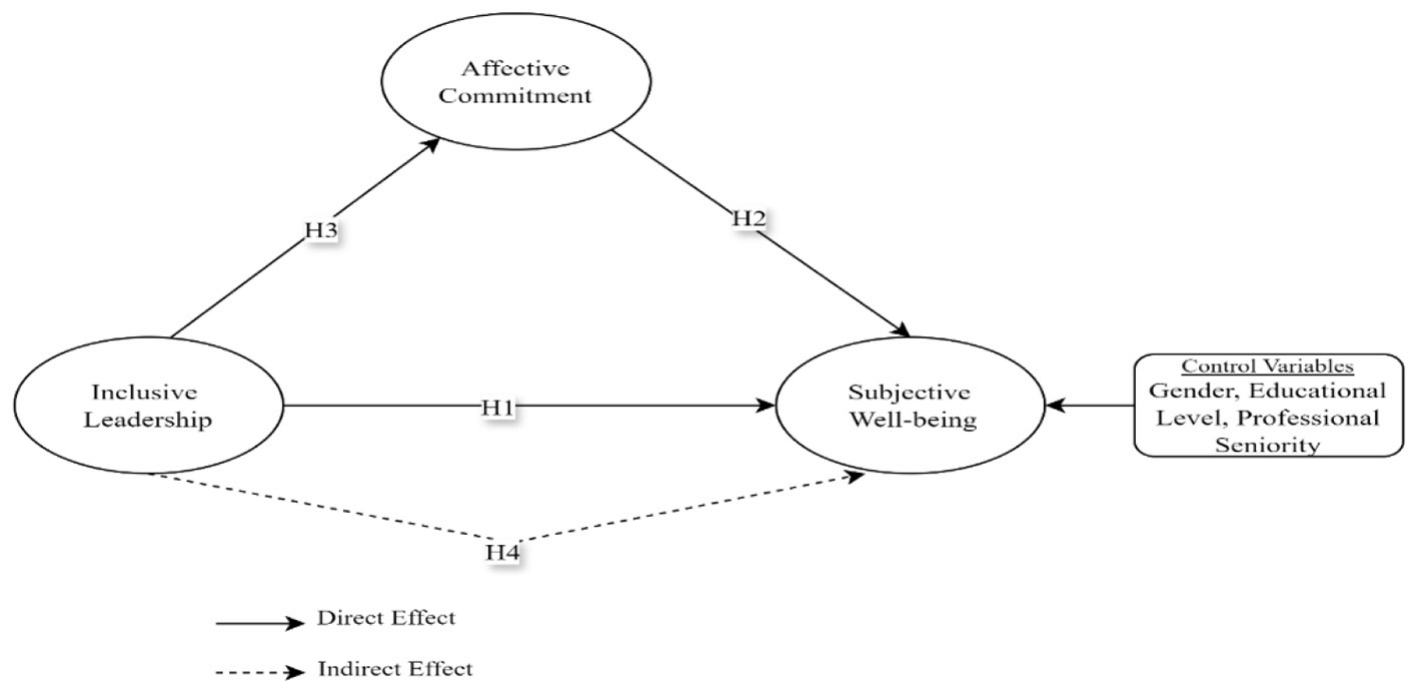
Conceptual model of the study.

The theoretical framework of the study can be based on Social Exchange Theory (Blau, 1964) and Self-Determination Theory (Deci and Ryan, 1985). According to Social Exchange Theory, individuals develop behavior based on the principle of reciprocity in their social relationships. Accordingly, as teachers feel supported in line with inclusive leadership approaches, their affective commitment to the institution and subjective wellbeing levels increase (Cropanzano and Mitchell, 2005). The Self-Determination Theory, on the other hand, argues that an individual's psychological wellbeing depends on the fulfillment of three basic needs: autonomy, competence, and relatedness (Ryan and Deci, 2000). Inclusive leadership strengthens teachers' intrinsic motivation and increases their subjective wellbeing by supporting these needs. These two theories complement each other in that Social Exchange Theory highlights the reciprocal and relational processes that link leadership behaviors with teachers' attitudes, while Self-Determination Theory focuses on how these relational conditions support intrinsic motivation through the fulfillment of basic psychological needs. Considering them together provides a more comprehensive understanding of how inclusive leadership is associated with both affective commitment and subjective wellbeing. When Social Exchange Theory and Self-Determination Theory are considered together, it can be seen that inclusive leadership supports teachers' wellbeing by meeting their intrinsic needs and reinforces their affective commitment through reciprocal social exchange processes. Therefore, the present study was modeled based on Social Exchange Theory and Self-Determination Theory (see Figure 1).

### 1.2 Literature review and research hypotheses

#### 1.2.1 The relationship between inclusive leadership and teachers' subjective wellbeing

Inclusive leadership is a leadership approach that aims to create an organizational climate in which managers establish trust-based relationships with their employees by demonstrating openness, accessibility and approachability; taking individual needs and expectations into account; allowing employees to freely express their thoughts; and ensuring psychological safety (Carmeli et al., 2010; Nembhard and Edmondson, 2006). Inclusive leadership stands out as a sustainable form of leadership that takes cultural diversity into account and respects differences in teaching and learning processes (Read et al., 2016). Subjective wellbeing is defined as an individual's positive assessment of their life and experiences, high satisfaction with life, and experiencing positive emotions more frequently and intensely than negative emotions (Diener et al., 2002). In this study, teachers' subjective wellbeing was examined in terms of “school commitment” and “teaching competence.” School commitment refers to feelings of support and relational belonging, while teaching competence is based on teachers' belief in their own professional competence and self-efficacy (Renshaw et al., 2015). The literature contains findings indicating that inclusive leadership practices contribute to the creation of a more inclusive climate in schools and increase teachers' subjective wellbeing (Abdi et al., 2023; Cao et al., 2022; Choi et al., 2017; Wang et al., 2021). In this regard, inclusive leadership provides a structure that supports both the individual and professional quality of life of teachers. On the other hand, alleviating teachers' negative working conditions makes them feel better about themselves (Windlinger, 2021) and increases their tendency to maintain emotional commitment (Leithwood and Mascall, 2008). In this context, it is thought that inclusive leadership can make positive contributions to teachers' subjective wellbeing. The first hypothesis of the study in this direction is stated as follows:

H:1 Inclusive leadership is positively and significantly associated with teachers' subjective wellbeing.

#### 1.2.2 The relationship between affective commitment and teachers' subjective wellbeing

Affective commitment refers to an employee's strong sense of belonging to an organization, identification with its goals and values, and a desire to contribute sincerely to the organization's success (Allen and Meyer, 1990). In the context of education, affective commitment is related to teachers' intrinsic commitment to their professional roles and institutions (Xu and Pang, 2024). Affective commitment gains further meaning in educational organizations due to the emotionally charged nature of teaching (Çelik et al., 2024). This is because teachers' affective commitment to their profession and the institution they work for is critical to the sustainability and quality of educational processes (Shu, 2022). In addition, affective commitment is not limited to institutional outputs but also emerges as a factor that directly affects teachers' subjective wellbeing (Amoah et al., 2021). In this regard, affective commitment increases teachers' subjective wellbeing, reduces professional burnout, and increases job satisfaction (Benevene et al., 2018). Studies in the literature also support the relationship between affective commitment and subjective wellbeing (Amoah et al., 2021; Li et al., 2024; Wang et al., 2021) and teachers' affective commitment is considered one of the key factors that strengthen their subjective wellbeing and ensure sustainability in education (El Kalai et al., 2022). Within the framework of these conceptual and empirical findings, the second hypothesis of the study is determined as follows:

H2: Affective commitment is positively and significantly associated with teachers' subjective wellbeing.

#### 1.2.3 The relationship between inclusive leadership and affective commitment

Inclusive leaders‘ openness toward their subordinates and willingness to evaluate new ideas enable employees to feel respected and that their efforts are appreciated (Carmeli et al., 2010). These positive perceptions strengthen employees' affective commitment to the organization (Zhu et al., 2009). The accessibility and supportiveness of inclusive leaders contribute to employees‘ perception of the organization as a reliable environment (Qi et al., 2019). By appreciating employees and enabling them to contribute, inclusive leaders strengthen their sense of belonging, helping them identify with the organization and develop affective commitment (Randel et al., 2018). Various research results in the literature (Cai et al., 2018; Kim and Sung, 2024; Ly, 2023) reveal that inclusive leadership has a significant and positive effect on employees' affective commitment to the organization. Therefore, based on the findings in the literature, the third hypothesis of the study is formulated as follows:

H3: Inclusive leadership is positively and significantly associated with affective commitment.

#### 1.2.4 The mediating role of affective commitment in the relationship between inclusive leadership and teachers' subjective wellbeing

The literature emphasizes that inclusive leadership has a significant impact on teachers‘ subjective wellbeing (Liu et al., 2024; Umrani et al., 2024). The openness, accessibility, and availability demonstrated by school administrators in their inclusive leadership approaches increase teachers' satisfaction with their interactions with administrators (Choi et al., 2017). This contributes to an increase in teachers‘ subjective wellbeing levels (Cao et al., 2022). Studies conducted in educational organizations also show that different types of leadership are related to teachers' subjective wellbeing (Cann et al., 2021; Dreer, 2023). However, since teaching is an emotionally intense and demanding profession, it is stated that this relationship may occur on the basis of affective commitment (Berkovich and Eyal, 2015). Some studies (Choi et al., 2015; Wang et al., 2020) have also found that inclusive leadership has a positive and significant relationship with affective commitment, indicating that the effect of inclusive leadership on subjective wellbeing may occur through affective commitment. In line with this conceptual and empirical framework, the final hypothesis of the study is as follows:

H4: The relationship between inclusive leadership and teachers' subjective wellbeing is associated with affective commitment.

## 2 Method

This study was designed as a cross-sectional and correlational model in line with the quantitative approach. Cross-sectional correlational models aim to examine the relationships between variables within a specific time frame (Cohen et al., 2013). The cross-sectional and correlational model was preferred in this study because the data was collected within a specific time frame and the relationships between variables were examined without seeking causality. On the other hand, scales were used in the study because data collection could be carried out in a short time and at low cost (Connelly, 2016). This section consists of the following headings: participants and data collection process, variables and data collection tools, and data analysis.

### 2.1 Participants and data collection process

The participants of this study consist of 585 teachers working in different educational levels and public schools in the western, eastern, northern, and southern provinces of Türkiye. The participants were selected using a convenience sampling method, and the provinces where the study was conducted were carefully selected to represent the socio-economic and cultural diversity of their regions. However, since the study was designed as a single-level analysis focusing only on teachers, no school- or region-level variables were collected, and no comparisons were made between regions or school types. The main goal was to reach a diverse teacher group across Türkiye rather than to test regional differences. Convenience sampling was preferred due to time and access constraints, as it provided a quick and practical way to collect data with limited resources (Etikan et al., 2016). In the data collection process, teachers were first reached through school administrators, and the 3 month data collection process was conducted online. The scale forms were sent to participants via email and WhatsApp. This method was preferred because it was efficient in terms of time and cost and provided the opportunity to reach a wide sample (Ponchio et al., 2021). However, we acknowledge that online distribution methods may favor participants with higher levels of digital accessibility and familiarity with online communication tools. To minimize this risk, school administrators directly shared the survey link with all teachers in their institutions, including those who may be less active users of digital platforms. In addition, the broad demographic variation observed in the final sample (gender, school level, education level, and professional seniority) suggests that a diverse cross-section of teachers was successfully reached despite the online distribution format. Participants filled out the forms anonymously and voluntarily. A total of 710 teachers were reached, and 595 teachers completed the data collection with a response rate of approximately 84%. However, the data of 10 participants were excluded as outliers, and the analyses were conducted on 585 teachers. These details regarding the total number of teachers reached, valid responses, and data cleaning process have been added to improve transparency. Additionally, to reduce social desirability and method bias, the scale form was structured according to the dependent, mediating, and independent variable order (MacKenzie and Podsakoff, 2012). Of the participants, 345 (59%) were male teachers and 240 (41%) were female teachers. In terms of educational level, 181 (30.9%) teachers work in high schools, 156 (26.7%) in middle schools, 151 (25.8%) in primary schools, 43 (7.4%) in pre-school institutions, and 54 (9.2%) in other institutions. In terms of educational level, 492 teachers (84.1%) hold a bachelor's degree, while 93 teachers (15.9%) hold a postgraduate degree. The average professional seniority of the participants is 15.22 (SD = 8.58). This study was conducted by administering online surveys with the informed consent of the participants. The ethics committee of Siirt University reviewed and approved the study. The official approval number is 8,121. To ensure data confidentiality, no identifying information was collected, IP addresses were not recorded by the online survey system, and all responses were stored anonymously without linking any personal or digital identifiers to participants.

### 2.2 Variables and data collection tools

*Independent Variable: Inclusive Leadership Scale (ILS)*. ILS was developed by Carmeli et al. (2010) and adapted into Turkish by (Gül and Çakici 2021). The ILS is a unidimensional, 9-item likert-type measurement tool (example item: My manager is willing to consult on issues) rated on a scale ranging from 1 (Strongly disagree) to 5 (Strongly agree). For this study, the results of the confirmatory factor analysis (CFA) of the scale were found to be statistically appropriate (*x*^2^/sd = 3.21; RMSEA = 0.062, SRMR = 0.027, CFI = 0.98, TLI = 0.97). The Cronbach's alpha (α) reliability coefficient of the scale was found to be 0.91; the average variance explained (AVE) was 0.52; and the composite reliability (CR) values were 0.90. The square root of the AVE value for the inclusive leadership scale (√0.52 = 0.72) is greater than its correlations with the other constructs (Affective Commitment: r = 0.685; Subjective Wellbeing: r = 0.527). In addition, the HTMT ratios were found to be below the recommended threshold of 0.85 (with Affective Commitment: 0.78; with Subjective Wellbeing: 0.62). Based on the criteria proposed by Fornell and Larcker (1981) and the HTMT results (Henseler et al., 2015), these findings indicate that the inclusive leadership scale demonstrates adequate discriminant validity.

*Instrument Variable: Affective Commitment Subscale (ACS)*. ACS is a subscale of the Organizational Commitment Scale developed by Meyer et al. (1993). The Turkish adaptation of the organizational commitment scale was made by Dagli et al. (2018). ACS consists of 6 items (example item: This school has a very special place for me) and is answered on a Likert scale ranging from 1 (Strongly disagree) to 5 (Strongly agree). Within the scope of the current study, the CFA results of the subscale were found to be statistically appropriate (*x*^2^/sd = 1.40; RMSEA = 0.026, SRMR = 0.014, CFI = 0.99, TLI = 0.99). Additionally, the Cronbach alpha (α) reliability coefficient of the subscale was calculated as 0.76, the average variance explained (AVE) as 0.59, and the composite reliability (CR) values as 0.73. The square root of the AVE value for the affective commitment scale (√0.59 = 0.77) is greater than the correlations of the affective commitment construct with the other constructs (Inclusive Leadership: r = 0.685; Subjective Wellbeing: r = 0.456). In addition, the HTMT ratios were found to be below the recommended threshold of 0.85 (with Inclusive Leadership: 0.77; with Subjective Wellbeing: 0.58). According to the Fornell and Larcker (1981) criteria and the HTMT results (Henseler et al., 2015), these findings indicate that the affective commitment scale demonstrates adequate discriminant validity.

*Dependent Variable: Subjective Wellbeing Scale (SWBS)*. The two-dimensional, eight-item SWBS, originally developed by Renshaw et al. (2015) and translated into Turkish by (Ergün and Nartgün 2017), was used to measure teachers' subjective wellbeing levels. The scale is a four-point Likert-type measurement tool with responses ranging from 1 (Almost never) to 4 (Almost always). The scale consists of two dimensions: school commitment (e.g., “I feel that I belong to this school”) and teaching competence (e.g., “As a teacher, I have achieved many things”), each comprising four items. For this study, it was determined that the CFA results of the scale were statistically appropriate (*x*^2^/sd = 3.74; RMSEA = 0.069, SRMR = 0.041, CFI = 0.97, TLI = 0.96). Additionally, the Cronbach's alpha (α) reliability coefficients for the scale were calculated as 0.82, 0.79, and 0.84 for the school commitment, teaching competence dimensions, and the entire scale, respectively; the AVE values were 0.54, 0.51, and 0.55; and the CR values were 0.77, 0.80, and 0.88. The square root of the AVE value for the subjective wellbeing scale (√0.55 = 0.74) is greater than the correlations of the subjective wellbeing construct with the other constructs (Inclusive Leadership: r = 0.527; Affective Commitment: r = 0.456). In addition, the HTMT ratios were found to be below the recommended threshold of 0.85 (with Inclusive Leadership: 0.62; with Affective Commitment: 0.56). According to the Fornell and Larcker (1981) criteria and the HTMT results (Henseler et al., 2015), these findings indicate that the subjective wellbeing scale demonstrates adequate discriminant validity.

*Control Variables:* Empirical evidence in the literature indicates that demographic variables are associated with subjective wellbeing (e.g., Fu et al., 2022; Özcan, 2024; Sagita et al., 2025). Therefore, gender, education level, and professional seniority variables were analyzed as control variables in the study.

### 2.3 Data analysis

All analyses in this study were conducted using AMOS 24 and SPSS 25 software. CFA and structural equation model (SEM) results were obtained using AMOS 24, while descriptive statistics and Pearson correlation analyses were performed using SPSS 25.00 analysis software. SEM was chosen because it allows for the simultaneous analysis of direct and indirect associations between observed and latent variables and is widely recommended for evaluating complex mediation models (Kline, 2015; Schumacker and Lomax, 2016). Prior to the analysis, assumption checks were performed on the data set; responses from a total of 10 participants with Z values exceeding ±3 and Mahalanobis distances exceeding the critical chi-square value were excluded, and the analyses were conducted on 585 data points. The Skewness (-0.426,−0.179) and Kurtosis (-0.695, 0.414) values of the data being within the ±1.5 limits indicate that univariate normality is achieved (Tabachnick and Fidell, 2013). Additionally, the data's proximity to the 45° reference line in the Q-Q plots supports normality. Multivariate normality was confirmed through multivariate scatter plots. Therefore, all these results indicate that the dataset meets the necessary assumptions for analysis. On the other hand, Pearson correlation coefficients between independent variables being below 0.80 (see Table 1), the VIF value being 1.884 (< 5), the condition index (CI) values (15.007 and 21.171) < 30, the tolerance index (TI) (0.531)>0.20 indicate that there is no multicollinearity issue in the study (Kutner et al., 2020).

**Table 1:** Descriptive statistics and Pearson correlation results (*n* = 585).

**Variables**	***M***	***SD***	**1**	**2**	**3**	**4**	**5**	**6**
1. Inclusive Leadership	3.78	0.61	-					
2. Affective Commitment	3.71	0.52	0.685^**^	-				
3. Subjective Wellbeing	3.22	0.46	0.527^**^	0.456^**^	-			
4. Gender	-	-	0.006	0.078	0.004	-		
5. Level of Education	-	-	−0.054	−0.020	0.002	0.037	-	
6. Professional Seniority	-	-	0.044	0.065	0.205^**^	0.072	0.079	-

^**^p < 0.01, M, Mean; SD, Standard Deviation; Gender: Reference group is male, Education Level: Reference group is undergraduate.

Since collecting data from a single source and within the same time frame may increase the risk of common method bias, Harman's single factor test was applied to test this situation (Harman, 1967). The results of the varimax rotated factor analysis conducted with all items belonging to the three variables indicate that a single factor explains 37.329% of the variance, suggesting that common method bias does not pose a serious threat. Additionally, the latent method factor approach, a more advanced method for common method bias, was also employed in the study. In this analysis, all items were loaded simultaneously onto both their theoretical constructs and a second latent common method factor (Williams et al., 1989). When examining the structural validity of the model, it was determined that the method factor did not contribute significantly to the model's fit indices and did not cause a serious deviation exceeding 0.20% in the factor loadings of the original items. These results also indicate that there was no serious common method bias problem in the self-report-based data used in the study. Direct and indirect associations were examined using bootstrapping analysis conducted with a sample size of 5,000 within the research model. The absence of zero within the 95% confidence intervals indicates that these associations are statistically significant (Preacher and Hayes, 2008). The validity of the scales was evaluated using various fit indices within the CFA framework. x^2^/sd ≤ 3 indicates good fit, while ≤ 5 indicates acceptable fit. CFI and TLI values between 0.95–1.00 indicate good fit, while values between 0.90–0.95 indicate acceptable fit (Hu and Bentler, 1999). RMSEA and SRMR values ≤ 0.05 indicate good fit, ≤ 0.08 acceptable fit, and ≥0.10 poor fit (Schermelleh-Engel et al., 2003). The reliability and validity of the scales were evaluated based on Cronbach's Alpha (α ≥ 0.70), composite reliability (CR ≥ 0.70), average variance explained (AVE ≥ 0.50), and CR > AVE criteria (Fornell and Larcker, 1981). Additionally, The VAF (Variance Accounted For) value was calculated to determine whether the mediating association was partial or full. VAF value below 0.20 indicates no mediating effect, a value between 0.20 and 0.80 indicates a partial mediating effect, and a value of 0.80 or above indicates a full mediating effect (Hair et al., 2017). The magnitude of the Beta (β) coefficients is interpreted as low at 0.10, moderate at 0.30, and high at 0.50 or above (Kline, 2015).

### 2.4 Descriptive statistics and correlation findings

Prior to analysis, the arithmetic mean and standard deviation values of the variables were calculated, and Pearson correlation coefficients were examined to evaluate the relationships between the variables (see Table 1).

As shown in Table 1, teachers' perceptions of inclusive leadership are high, with an average of 3.78 (SD = 0.61). Their levels of affective commitment were also found to be similarly high (M = 3.71, SD = 0.52). Teachers' subjective wellbeing levels, however, are moderate (M = 3.22, SD = 0.46). In terms of standard deviation values, teachers' views on inclusive leadership (SD = 0.61) differ more than their views on affective commitment (SD = 0.52) and subjective wellbeing (SD = 0.46). According to the results of Pearson correlation analysis, a positive and significant association was found between inclusive leadership and affective commitment (r = 0.685, *p* < 0.01) and subjective wellbeing (r = 0.527, *p* < 0.01). In addition, a positive and significant relationship was found between affective commitment and subjective wellbeing (r = 0.456, *p* < 0.01) and between professional seniority and subjective wellbeing (r = 0.205, p < 0.01). No significant associations were found between the other demographic variables (gender and education level) and the main variables.

### 2.5 Findings regarding hypotheses

**Figure 2:**
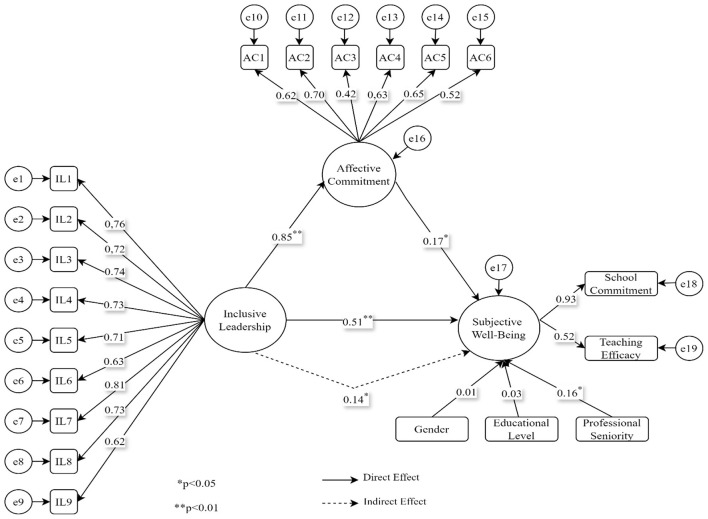
SEM analysis results of the study.

The hypotheses developed in the study were tested through statistical analyses following a systematic method. In this context, the associations among variables were evaluated in a multifaceted manner, and the analysis process was based on testing each hypothesis (H1, H2, H3, and H4) separately. Detailed results regarding the hypotheses of the study are provided (see Figure 2 and Table 2).

**Table 2:** Standardized path coefficients for direct and mediating effects in the model (*n* = 585).

**Type of Impact**	**Independent variable**	**Agent variable**	**Dependent variable**	**Direct and indirect effects**		
				β	**SE**	**95% CI [LCI-UCI]**
Direct impact	Inclusive leadership	-	Subjective wellbeing	0.51^**^	0.119	[0.287 0.750]
	Affective commitment	-	Subjective wellbeing	0.17^*^	0.114	[0.078 0.393]
	Inclusive leadership	-	Affective commitment	0.85^**^	0.077	[0.804 0.899]
	Gender	-	Subjective wellbeing	0.01	0.040	[-0.070 0.072]
	Level of education	-	Subjective wellbeing	0.03	0.054	[-0.043 0.093]
	Professional seniority	-	Subjective wellbeing	0.16^*^	0.041	[0.092 0.252]
Indirect impact	Inclusive leadership	Affective commitment	Subjective wellbeing	0.14^*^	0.110	[0.074 0.330]

^*^p < 0.05, ^**^p < 0.01, SE, Standardized Error; CI, Confidence Interval; LCI, Lower Confidence Interval; UCI, Upper Confidence Interval; Gender: Reference group is male, Education Level: Reference group is undergraduate (bachelor's degree).

**Figure 3:**
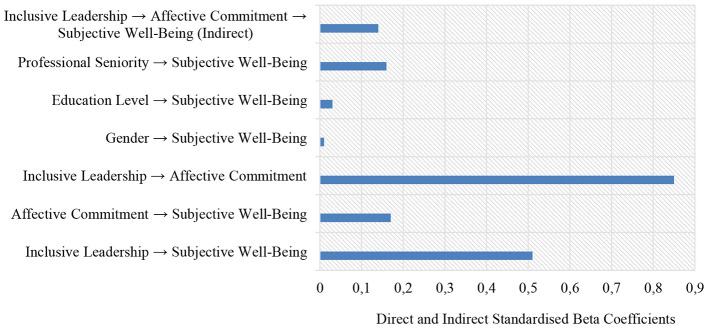
Effect sizes related to standardized coefficient values.

The fit values of the structural equation model created in Figure 2 were found to be within appropriate ranges (*x*^2^/sd = 3.46; RMSEA = 0.065, SRMR = 0.048, CFI = 0.92, TLI = 0.91) (Hu and Bentler, 1999; Schermelleh-Engel et al., 2003), indicating that the proposed model demonstrates an acceptable level of fit. As seen in Figure 2 and Table 2, the association between inclusive leadership and subjective wellbeing was positive and significant (β = 0.51, SE = 0.119, 95% CI [0.287 0.750]). Similarly, the association between affective commitment and subjective wellbeing was found to be positive and significant, although weaker in magnitude (β = 0.17, SE = 0.114, 95% CI [0.078 0.393]). In addition, inclusive leadership was strongly and positively associated with affective commitment (β = 0.85, SE = 0.077, 95% CI [0.804 0.899]). Furthermore, *R*^2^ values were calculated to demonstrate the proportion of variance explained in the dependent variables within the structural equation model. The *R*^2^ value for the affective commitment variable was found to be 0.72, suggesting that inclusive leadership is associated with a substantial proportion of the variance in affective commitment, rather than implying causality. For the subjective wellbeing variable, inclusive leadership, affective commitment, and the control variables (gender, educational level, and professional tenure) jointly accounted for 32% of the variance (*R*^2^ = 0.32). This value suggests that the structural model possesses a moderate level of explanatory power. When indirect associations are examined, the association between inclusive leadership and subjective wellbeing through affective commitment was significant but low in magnitude (β = 0.14, SE = 0.110, 95% CI [0.074 0.330]). At the same time, the VAF≈22% value obtained in this study is evaluated within the scope of the partial mediation criterion (Hair et al., 2017). Thus, affective commitment can be interpreted as partially associated with the linkage between inclusive leadership and subjective wellbeing. These results suggest that H1, H2, H3, and H4 are supported. Regarding the control variables, gender was not significantly associated with subjective wellbeing (β = 0.01, SE = 0.040, 95% CI [-0.070 0.072]). Education level was also not significantly associated with subjective wellbeing (β = 0.03, SE = 0.054, 95% CI [-0.043 0.093]). Professional seniority showed a low but significant association with subjective wellbeing (β = 0.16, SE = 0.041, 95% CI [0.092 0.252]). Overall, the findings show that inclusive leadership is positively associated with subjective wellbeing, both directly and through affective commitment, whereas the associations involving the control variables are limited. Finally, to examine whether the structural model operated equivalently across gender groups, measurement and structural invariance analyses were conducted using a multi-group structural equation modeling approach. In this framework, a sequence of progressively constrained models was tested for female and male participants: configural invariance, metric invariance, scalar invariance and strict invariance. Since the difference in CFI values between the nested models (ΔCFI) was below the recommended threshold of 0.01 (Hu and Bentler, 1999), invariance was supported at each level. These results indicate that the structural model functions in an equivalent manner for both female and male groups. Figure 3 shows the effect sizes of the standardized coefficient values (see Figure 3).

## 3 Discussion and conclusion

This study found a positive and significant association between inclusive leadership and teachers' subjective wellbeing. The literature indicates that school principals who demonstrate inclusive leadership, along with openness, accessibility, and valuing teachers' opinions, are associated with lower levels of perceived job stress and are related to subjective wellbeing levels (Choi et al., 2017; Edmondson et al., 2016). In this context, teachers' participation in school management in line with inclusive leadership practices appears to be associated with strengthened subjective wellbeing (Abdi et al., 2023). The findings suggest that inclusive leadership practices, in which teachers' needs are taken into account and they are effectively involved in decision-making processes in the school environment, are associated with higher levels of subjective wellbeing. Similarly, inclusive leadership has been found to be associated with employees' subjective wellbeing through increased job adaptation (Choi et al., 2017), to be related to enhanced work and personal resources among employees with disabilities (Luu, 2019), and to be linked with improved perceptions of subjective wellbeing in inclusive and supportive workplace climates (Cao et al., 2022). Beyond confirming these associations, the findings of this study offer new insights by showing that inclusive leadership operates as a compensatory resource within Türkiye's highly centralized and bureaucratic educational system. In contexts where rigid structures limit teachers' professional autonomy, supportive and participatory leadership behaviors play a crucial role in meeting teachers' psychological needs and enhancing wellbeing (Hallinger and Hammad, 2020; Arar and Oplatka, 2022; Bellibaş et al., 2023). From a conceptual perspective, inclusive leadership is also discussed in relation to sustainable education, highlighting its potential contribution to a more equitable and supportive school climate rather than presenting sustainability as a tested construct. This study provides a unique contribution by examining the associations between inclusive leadership and teachers' subjective wellbeing within Türkiye's centralized and bureaucratic school system. From a conceptual perspective, inclusive leadership is also discussed in relation to sustainable education, highlighting its potential contribution to a more equitable and supportive school climate rather than presenting sustainability as a tested construct. In this conceptual framing, sustainable education refers not only to environmental considerations but also to the social dimension of sustainability, which includes equity, participation, teachers' professional wellbeing and the development of supportive organizational climates (Barth et al., 2015; Rogozińska-Pawełczyk, 2023). Inclusive leadership contributes to this social dimension through practices that encourage participatory decision-making, value diversity, and strengthen teachers' psychological safety, and sense of belonging. These elements are important for maintaining healthy and resilient school environments over time (Brimhall and Mor Barak, 2018; Ye et al., 2019). In highly centralized systems such as Türkiye, where rigid governance structures may restrict professional autonomy, inclusive leadership becomes particularly significant in supporting the human and relational aspects of sustainable education. It should also be noted that all variables in this study were measured through self-report instruments administered at a single time point, which may naturally introduce method-related bias. However, to address this concern, a latent method factor analysis was conducted as part of the measurement validation process (Williams et al., 1989). The results showed that the method factor did not meaningfully improve model fit and did not cause substantial distortions in factor loadings, indicating that method-related bias was minimal in this dataset. Additionally, the questionnaire was structured by ordering the dependent, mediating, and independent variables separately, following the recommendations of MacKenzie and Podsakoff (2012), which is another procedural technique suggested for reducing method-related bias.

The present study found a positive and significant association between affective commitment and teachers' subjective wellbeing. Other studies in the literature also support the positive association between affective commitment and teachers' subjective wellbeing (Amoah et al., 2021; Li et al., 2024; Wang et al., 2021). The nature of the teaching profession, which involves heavy workloads, constant interaction with students, and similar situations, requires a significant amount of emotional labor (Luna et al., 2022). In addition, factors such as daily excessive fatigue, difficulties encountered in classroom management, and time constraints can increase emotional demands and are associated with emotional exhaustion among teachers (Li et al., 2024). However, strengthening teachers' affective commitment by reducing their stress levels has been shown in previous literature to be associated with their subjective wellbeing (Liu et al., 2021). Previous studies have shown that affective commitment is associated with lower professional burnout, is related to job satisfaction, and is associated with higher levels of subjective wellbeing (Benevene et al., 2018). Furthermore, teachers' affective commitment, their perceptions of leadership practices, and their understanding of sustainable education have been identified as key factors that relate to their subjective wellbeing (El Kalai et al., 2022). Therefore, it is understood that the positive association between affective commitment and teachers' subjective wellbeing is consistent with the existing literature.

Another important finding of the study reveals that there is a positive and significant association between inclusive leadership and affective commitment. This result is consistent with findings from previous studies that examined similar associations (Cai et al., 2018; Choi et al., 2015; Kim and Sung, 2024; Ly, 2023; Xiaotao et al., 2018). Characteristics such as openness, accessibility, and availability demonstrated within inclusive leadership approaches are considered elements that meet teachers' needs to feel competent, connected, and in control of their work (Çelik et al., 2024; Wang et al., 2020). In this context, school administrators' higher levels of inclusive leadership behaviors are associated with higher levels of teachers' organizational commitment (Baş, 2022) and tend to be linked with stronger organizational commitment overall (Yasin et al., 2023). In line with previous conceptual work, the present study discusses inclusive leadership within the contextual framework of sustainable education, emphasizing diversity and participation as core principles of this context, rather than empirically examining sustainability as a variable. From a policy perspective, the findings suggest that inclusive leadership can be supported within Türkiye's highly centralized system by expanding school-level decision-making authority, strengthening principals' instructional autonomy, and providing national professional development programs that encourage participatory leadership practices. Prior research shows that bureaucratic governance structures in Türkiye often limit school leaders' capacity for autonomous decision-making, making inclusive and collaborative leadership approaches particularly valuable in such contexts (Ahmet, 2022; Bellibaş et al., 2023; Kilinç et al., 2021).

The final hypothesis of our study proposes that affective commitment is associated with the relationship between inclusive leadership and teachers‘ subjective wellbeing. Inclusive leadership is associated with higher levels of affective commitment and, consequently, greater subjective wellbeing by making teachers feel like an integral part of the school (Brimhall and Mor Barak, 2018; Shu, 2022). Affective commitment is related to internal belonging and dedication to the professional role and organization (Xu and Pang, 2024), is associated with higher levels of subjective wellbeing (Amoah et al., 2021; Li et al., 2024; Wang et al., 2021). Inclusive leaders tend to support the sense of belonging by appreciating and involving their employees in the process, thereby reinforcing the relationship between affective commitment and leadership (Randel et al., 2018). The literature shows that inclusive leadership is associated with affective commitment (Cai et al., 2018; Kim and Sung, 2024; Ly, 2023) and that this may occur through mediators in different leadership approaches (Donkor, 2021; Duarte et al., 2021; Saleem et al., 2020). However, while most studies consider psychological capital as an instrumental variable (Bouckenooghe et al., 2015; Fang et al., 2019; Wu and Nguyen, 2019), the present study makes a unique contribution to the literature by highlighting the association between inclusive leadership and teachers' subjective wellbeing and the potential mediating role of affective commitment.

The literature indicates that sustainable education is mostly addressed within the framework of environmental education, but sustainable leadership is becoming increasingly important in connection with the professional development of education managers (O'Flaherty and Liddy, 2018). Studies conducted in Türkiye reveal that sustainable leadership has been examined in various dimensions, such as institutional culture, organizational justice, organizational identification, scale development and adaptation studies, and theoretical evaluations (Çetin and Baş, 2021; Ertaş and Özdemir, 2021). Consistent with this body of work, the current study conceptually links inclusive leadership and affective commitment with sustainability principles in education, without empirically testing sustainability as a variable. In light of the data obtained from a large sample of teachers in Türkiye, the research findings suggest that school administrators' adoption of inclusive leadership is associated with higher levels of teachers' subjective wellbeing through affective commitment. These findings may offer useful insights for understanding how more inclusive and contextually sustainable school environments can be associated with enhanced teacher wellbeing, rather than making direct empirical claims about sustainability.

### 3.1 Theoretical implications

The findings of this study indicate that affective commitment is associated with the relationship between inclusive leadership and teachers' subjective wellbeing, which is consistent with the findings reported in the literature (Abdi et al., 2023; Choi et al., 2017; Edmondson et al., 2016) and supports the theoretical framework. The results suggest that inclusive leadership behaviors are associated with higher levels of teachers' subjective wellbeing through their participation in decision-making processes (Cao et al., 2022; Luu, 2019; Ye et al., 2019) and that affective commitment may function as an important mediating variable in this association (Brimhall and Mor Barak, 2018; Shu, 2022; Xu and Pang, 2024). Considering the findings in the literature that affective commitment is associated with higher levels of job satisfaction and subjective wellbeing (Amoah et al., 2021; Benevene et al., 2018; Li et al., 2024; Wang et al., 2021), this study aligns with theoretical models linking inclusive leadership, affective commitment, and subjective wellbeing in the context of education. Furthermore, the research adds a new contextual dimension to the sustainable leadership literature by highlighting the association between inclusive leadership and affective commitment within the framework of sustainable education in Türkiye, which appears to be related to teachers' subjective wellbeing (Çetin and Baş, 2021; Ertaş and Özdemir, 2021).

### 3.2 Practical conclusions

Research findings suggest that higher levels of inclusive leadership behaviors among school principals are associated with higher levels of teachers‘ affective commitment and subjective wellbeing (Baş, 2022; Yasin et al., 2023). Leadership practices such as transparency, accessibility, valuing employee opinions, and ensuring active participation in decision-making processes are associated with greater professional satisfaction and commitment to the school among teachers (Çelik et al., 2024; Wang et al., 2020). specially in the teaching profession, which is characterized by high workloads and intense emotional demands, supportive and inclusive approaches by leaders are associated with lower stress levels and reduced risk of burnout (Liu et al., 2021; Luna et al., 2022). From the perspective of education policymakers, it is recommended that inclusive leadership principles be integrated into manager training programmes and that leadership models that support sustainability in the school climate be disseminated. This would support both teachers' professional motivation and the construction of a sustainable educational environment.

### 3.3 Limitations of the study and recommendations for future research

This study was conducted using cross-sectional data collection methods, and although the associations between variables were found to be statistically significant, it is not possible to draw definitive conclusions about causality. Therefore, it is suggested that future studies use longitudinal designs to examine the associational patterns or potential causal directions between variables with stronger evidence. In addition to longitudinal designs, future research could also benefit from adopting mixed-methods approaches, which would allow quantitative findings to be complemented and enriched through qualitative insights. Furthermore, since the research was limited to teachers working in public schools, the generalisability of the results to private school teachers or employees in different educational institutions is limited. To overcome this limitation, future studies could increase diversity and representativeness by including private school teachers and participants from different branches and levels in the sample. The fact that the data was collected through self-reporting carries the risk of social desirability and common method bias. In this study, a latent method factor analysis was conducted during the measurement validation process, and the method factor did not meaningfully improve model fit or distort factor loadings, indicating that method-related bias was minimal. In addition, the structure of the questionnaire was designed by ordering the dependent variables, mediating variables, and independent variables in separate sections, in line with the procedural remedies suggested by MacKenzie and Podsakoff (2012), which is a commonly recommended method for reducing method-related bias. Nevertheless, the possibility of such bias cannot be entirely ruled out due to the single-time and self-reported nature of the data. To reduce this risk, it is recommended that future studies use qualitative data collection techniques such as observation, interviews, or focus groups to support the results. Although convenience sampling enabled access to a large and diverse group of teachers across Türkiye, it inherently limits the representativeness of the sample and may introduce sampling bias. Convenience sampling is widely acknowledged to reduce external validity because volunteer participants may systematically differ from the broader teacher population (Etikan et al., 2016; Bornstein et al., 2013). In addition, collecting data through online tools such as email and WhatsApp may have disproportionately included teachers with higher digital access or familiarity with technological communication platforms. Although school administrators supported the distribution of the survey link to all teachers, this potential limitation should still be considered when interpreting the findings. Therefore, the findings should be interpreted with caution, and future research is encouraged to use probabilistic sampling strategies to enhance representativeness. On the other hand, future studies using hierarchical/multilevel analyses to examine individual and organizational variables together will provide more comprehensive results. Finally, in future studies, different mediating variables such as psychological safety, job satisfaction, or organizational support can be added to the model to increase the explanatory power of the research.

## Data Availability

The raw data supporting the conclusions of this article will be made available by the authors, without undue reservation.
